# Selected Large-Animal Models of Ventricular Arrhythmias

**DOI:** 10.3390/biology15040343

**Published:** 2026-02-15

**Authors:** Piotr Frydrychowski, Alicja Cepiel-Kośmieja, Zuzanna Wojtczak, Krzysztof Nowak, Agnieszka Noszczyk-Nowak

**Affiliations:** 1 Department of Internal Medicine and Clinic of Diseases of Horses, Dogs and Cats, Wrocław University of Environmental and Life Sciences, Grunwaldzki Sq. 47, 50-366 Wrocław, Poland; alicja.cepiel@upwr.edu.pl (A.C.-K.); zuzanna.sidoruk@upwr.edu.pl (Z.W.); 2 Institute of Heart Diseases, Wrocław Medical University, Borowska Str. 213, 50-556 Wrocław, Poland; krzysztof.nowak@umw.edu.pl

**Keywords:** large-animal models, ventricular arrhythmias, pigs, dogs, cats, sheep, goats

## Abstract

Ventricular arrhythmias are among the most life-threatening cardiac rhythm disturbances. Owing to their complex pathophysiology and high mortality risk, they remain a major focus of research aimed at elucidating underlying mechanisms and improving prevention and therapeutic strategies. In this context, animal models—particularly large-animal models—are of pivotal importance because they more closely recapitulate human cardiac anatomy and electrophysiology. The most commonly used species include dogs, cats, pigs, sheep and goats. Dogs have historically played a prominent role in ventricular arrhythmia research; however, their use is increasingly constrained by legal regulations and ethical–societal considerations. Sheep and goats, although employed less frequently, have also contributed meaningfully to advances in the field. Among large-animal models, swine are regarded as especially promising, largely due to the similarity between porcine and human hearts and the feasibility of reliably inducing ventricular arrhythmias, particularly under conditions of acute ischemia associated with coronary artery occlusion. Large-animal models are also indispensable for the preclinical evaluation of novel drugs, therapeutic approaches, and medical devices prior to translation into human studies. In this article, we review selected investigations employing large-animal models of ventricular arrhythmias, with particular emphasis on the materials and methods reported in the cited literature.

## Simple Summary

Ventricular arrhythmias represent a leading cause of sudden cardiac death and remain a critical challenge in cardiovascular medicine. Their multifactorial mechanisms and substantial fatality risk continue to drive intensive research focused on clarifying arrhythmogenic pathways and optimizing preventive and therapeutic approaches. Large-animal models are central to this effort because they more faithfully reflect human cardiac structure and electrophysiological properties than small-animal systems. Species most frequently used in this field include dogs, cats, pigs, sheep, and goats. Although canine models have contributed extensively to mechanistic and translational insights, their contemporary use is increasingly limited by regulatory constraints and ethical considerations. Ovine and caprine models, while less prevalent, provide complementary information and have supported progress in selected applications. Among available large-animal platforms, porcine models are widely considered the most translationally relevant, given the close anatomical and electrophysiological correspondence between pig and human hearts and the practicality of reproducibly inducing ventricular arrhythmias, particularly in acute ischemia following coronary occlusion. Importantly, large-animal studies remain indispensable for the preclinical assessment of emerging pharmacotherapies, interventional strategies, and medical devices prior to evaluation in humans. This article synthesizes representative studies using large-animal ventricular arrhythmia models, with a focus on reported materials and methodological approaches.

## 1. Introduction

Ventricular arrhythmias are life-threatening, and both basic and applied research are attempting to find answers on how to prevent their occurrence and how to treat them. The evaluation of the complex interplay between various cell types in the heart, including cardiomyocytes from the conduction system and the working myocardium, fibroblasts, and cardiac immune cells, remains a major challenge in arrhythmia research because it can be investigated only in vivo. Research on ventricular arrhythmias in animal models has long played an important role in understanding their mechanisms and developing treatment strategies. Without animal models, it would be impossible to understand many mechanisms of ventricular arrhythmias and develop effective pharmacological and non-pharmacological treatments. In studies of ventricular arrhythmias, large-animal models offer many advantages over small-animal models. A major limitation of small-animal models is that their fast heart rate, small heart size, and differential ionic currents do not fully recapitulate human cardiac electrophysiology. Although large-animal models obviously have their limitations, their role in research cannot be overestimated. Dogs have traditionally been widely used in arrhythmia research, but legal restrictions, societal considerations, and the lack of genetically engineered models restrict their future use. On the other hand, many naturally occurring diseases in dogs involve ventricular arrhythmias and may therefore serve as an important research model [1]. Swine models could close this translational gap because their use is more accepted in modern societies, pig cardiac anatomy and electrophysiology are similar to those of humans, and pigs can be genetically modified. On the other hand, new results suggest that, in the pig, the mean electrical axis is influenced more by the presence of an intramural Purkinje fiber network than by left ventricular mass, as seen in humans [2]. It is important to caution against extrapolating the results of electrophysiological or ventricular arrhythmia studies from porcine models to humans. Regardless, large animals are useful for preclinical studies evaluating new drugs, gene therapy, and devices that require large-animal models before moving to humans.

The diversity of species used in research and the variety of models prompted us to systematize our knowledge on this subject and present, in this review, the existing large-animal models of ventricular arrhythmias, divided by species and type.

## 2. Materials and Methods

### 2.1. Search for Literature to Be Included in This Review

Eligibility criteria for studies to be included in this review were: (1) including original data on a large-animal model of ventricular arrhythmias, (2) involving both natural and experimental models, (3) peer-reviewed published studies.

Exclusion criteria were: (1) studies that only include data on in vitro models, (2) studies on other species, (3) opinion pieces, (4) clinical studies of dogs and cats, (5) case studies. Data from non-peer-reviewed published studies, such as those presented at international conferences and published as proceedings, were not included in the search or in this review.

PubMed search was conducted with the following search words: ventricular arrhythmias combined with large-animal models, pig, porcine, horse, dog, cat, sheep, goats. In addition, PubMed was searched for the names of ventricular arrhythmias known to the authors, combined with pig, porcine, horse, dog, cat, sheep, and goat: ventricular tachycardia (VT) and ventricular fibrillation (VF). The last PubMed search was on 2 December 2025. The PubMed search and selection of studies to be included was conducted by all authors, excluding the senior author, and was double-checked by the senior author.

### 2.2. Data Retrieved from Literature and Data Analysis

A list of selected large-animal models of ventricular arrhythmias identified in the literature was compiled. Studies were grouped according to the species that was described. For each study, the following was reported: the type and characteristics of the model’s performance, and the model’s advantages and disadvantages.

## 3. Results

Through the PubMed search, 5655 studies were identified, of which 5570 were excluded for reasons described in the exclusion criteria, and 85 were included in this review. Large-animal models of ventricular arrhythmias identified through the literature search include several types: species-specific, ischemic, non-ischemic, with a natural course, and fully experimental. Table 1 summarizes the principal strengths and limitations of animal models employed in studies of ventricular arrhythmias.

### 3.1. Pig Model

Pigs are a good animal model for studying cardiac arrhythmias, especially ventricular arrhythmias. This is due to similarities between the pig and human hearts in anatomy, physiology, and electrophysiology, including heart size, hemodynamic parameters, myocardial electrophysiological properties, and coronary circulation anatomy. In addition, these animals are particularly susceptible to the development of ventricular arrhythmias, including ventricular fibrillation (VF) [3], which is an undeniable advantage of using pigs as an animal model for ventricular arrhythmia research.

There are no porcine models with a natural course of ventricular arrhythmias.

Experimental studies using pigs as an animal model for ventricular arrhythmias have used two main methods of inducing these arrhythmias: ischemic and non-ischemic. Ischemic models are usually induced by occluding one of the coronary arteries, intended to mimic the conditions that often lead to the development of ventricular arrhythmias in humans. Non-ischemic models, on the other hand, use various techniques such as rapid pacing, cardioneuroablation, or the administration of specific drugs to induce ventricular arrhythmias without the need for coronary artery occlusion.

#### 3.1.1. Pig Ischemic Model

In the ischemic model, the coronary artery is ligated or occluded. Early ischemic models were usually achieved by ligating the coronary vessels via a sternotomy approach. These techniques were highly invasive, but allowed observation of the impact of coronary vessel occlusion on the development of arrhythmias. Muller et al. [4] induced acute myocardial ischemia by ligating the anterior descending artery. Access to the heart was obtained via median sternotomy, while occlusion of the coronary vessels was achieved by tightening a silk ligature at the mid-length of the anterior descending artery. The researchers administered bucindolol (a β-blocker) intravenously twice, 30 min before and 10 min after coronary artery ligation, at a dose of 6 mg/kg, to evaluate its effect on ventricular arrhythmia induction. This drug significantly reduced the occurrence of VF and the duration of ventricular tachycardia (VT). Occlusion of the mid-segment of the left anterior descending artery (LAD) to induce ventricular arrhythmias was also used by Smith et al. [5]. After performing median sternotomy and suspending the heart in a pericardial cradle, the researchers ligated the LAD at its midportion for 60 min. This group demonstrated that arrhythmias of type Ib and electrocardiographic changes observed during ischemic episodes show a strong correlation with impaired intercellular electrical coupling. One of the more recent studies in which ischemia was induced by ligating the LAD after median sternotomy is the experiment by Howard-Quijano et al. [6]. In this study, LAD occlusion was performed using a 4-0 Prolene suture, which was passed through a short segment of polyethylene tubing and used to occlude the LAD for 5 min. Electrophysiological measurements were taken at baseline, after 30 min of spinal cord stimulation (SCS) or sham procedure, as well as at the moment of LAD ligation and after 45, 90, and 300 s of occlusion, and throughout the entire 60 min reperfusion period. The scientists demonstrated that spinal cord stimulation attenuates excessive local sympathetic stimulation in the ischemic area of the myocardium, thereby reducing the induction of ventricular rhythm disturbances and improving cardiac function. This study demonstrated the translational potential of these models, particularly for further research on the protective effects of SCS on the myocardium.

Contemporary studies on ischemic models most commonly use transient occlusion of the coronary arteries to induce ischemia. The use of balloon catheters under fluoroscopic guidance is widespread, allowing precise control of ischemia duration and extent, resulting in a low-invasive procedure and high reproducibility. These models have enabled assessment of arrhythmia occurrence during ischemic periods and during reperfusion, during which, paradoxically, restoration of blood flow can induce arrhythmias [7]. A less invasive method of coronary artery occlusion has also allowed researchers to analyze the role of various pharmacological interventions in mitigating arrhythmias induced by ischemia and reperfusion. By administering potential therapeutic agents at different time points during the ischemia–reperfusion process, scientists can evaluate their effectiveness in preventing or reducing the occurrence of arrhythmias following an episode of myocardial ischemia. Dawkins et al. investigated the effect of exosomes secreted by cardiosphere-derived cells (CDCEXO) on the inducibility of arrhythmias after ischemia and reperfusion induced by LAD occlusion [8]. In this experiment, LAD occlusion was induced in its proximal one-third by inflating a standard balloon angioplasty catheter (TREK) for 90 min. The reperfusion period after ischemia lasted 8 weeks. The researchers demonstrated, among other findings, that administration of exosomes after the reperfusion period reduced the size of the post-infarction scar and the inducibility of ventricular arrhythmias, and may represent an alternative to conventional ablation in preventing recurrent ventricular tachyarrhythmias following an episode of myocardial ischemia. In another study, a porcine ischemic model was used to evaluate the efficacy of pulsed-field ablation (PFA) of the post-infarction scar, which was induced by balloon occlusion of the LAD lasting 60–90 min. PFA was delivered in a bipolar/biphasic fashion (2.0 kV/application; 5 trains delivered over 2.5 s). This study demonstrated the efficacy of PFA in penetrating post-infarction scars of various origins and highlighted the beneficial effect of QRS gating during ablation on the inducibility of ventricular arrhythmias during PFA [9]. LAD occlusion to induce acute myocardial infarction (AMI) and subsequent reperfusion was used in the study by Odenstedt et al. [10]. In this model, AMI was induced by occluding the LAD distal to the second diagonal branch for 45 min, using different techniques. For LAD occlusion, depending on the phase of the experiment, the following were used: a) a 10 mm long, over-the-wire coronary angioplasty balloon catheter; b) a support catheter (Guidant) with an outer diameter of 1.5 mm with a ligature applied to its tip; and c) both inflation of a balloon catheter and a ligature applied around the inflated balloon. In this experiment, ventricular arrhythmias were not specifically studied; however, the ischemic myocardial infarction (MI) model created by balloon occlusion of the LAD was later used by Odenstedt and various research groups in subsequent studies [11,12]. These experiments showed that before the onset of VF, there was an aggravation of T-wave inversion, which may represent aspects of ventricular repolarization (VR) relevant to arrhythmogenesis [11], and that spinal cord stimulation (SCS) is thought to have antiarrhythmic effects in spontaneous non-sustained ventricular tachycardia (NSVT) and sustained VT during the ischemia–reperfusion period, along with a reduction in repolarization alterations [12]. 

A key aspect of ischemic models using coronary artery occlusion via balloon catheters was the relatively high mortality rate of animals when the occluded vessel was the LAD [13,14]. Frydrychowski et al., by modifying the anesthesia protocol in pigs, developed an ischemic model using occlusion of the proximal LAD segment, characterized by 100% animal survival and high inducibility of ventricular arrhythmias, making this model useful for studies of ventricular arrhythmias in acute myocardial ischemia [15]. In this study, ischemia was induced by inflating a 3.0 × 10 mm angioplasty balloon to 6 atm. LAD occlusion was maintained for 30 min [15].

An alternative ischemia-induction protocol was used to examine how increased sympathetic drive and reduced vagal tone modulate the inducibility of ventricular arrhythmias during myocardial ischemia. In this porcine model, myocardial ischemia was induced by balloon occlusion of the mid–left anterior descending (LAD) coronary artery using a coronary angioplasty catheter. After balloon inflation, a suspension containing sterile normal saline and variable volumes of polystyrene microspheres (Polybead, 90 μm; Polysciences, Warrington, PA, USA) was delivered through the angioplasty catheter. These models were not designed to directly induce ventricular arrhythmias per se, but rather to elucidate mechanisms that may contribute to the occurrence of ventricular arrhythmias during myocardial ischemia in humans [16,17,18].

In a study by Ajijola et al., post-infarction neural remodeling in a patchy-scar MI model reorganized functional sympathetic innervation and increased dispersion of repolarization. Sympathetic stimulation also altered scar-related conduction, supporting a role for repolarization heterogeneity and abnormal propagation in post-infarct reentrant arrhythmogenesis under heightened sympathetic tone [16]. In another experiment, Yoshie et al. showed that early epicardial resiniferatoxin (RTX, activator of the TRPV1 channel) application can ablate TRPV1 (transient receptor potential cation subfamily V member 1) sensory afferents that sustain chronic sympathoexcitation after MI. Cardiac-restricted suppression of this pathway attenuated adverse remodeling and electrical heterogeneity, thereby reducing VT/VF vulnerability and stabilizing infarcted hearts [17]. Work by Salavatian et al. suggests that chronic MI remodels vagal afferent nociceptive signaling toward an inhibitory, potentially GABA-mediated phenotype, thereby reducing central vagal drive and vagal tone. This mechanism may contribute to post-MI parasympathetic dysfunction and increased susceptibility to ventricular arrhythmias and sudden death [18].

#### 3.1.2. Pig Non-Ischemic Model

Non-ischemic models enable the analysis of ventricular arrhythmias in the absence of ischemia. The first models of this type were developed in the 1980s, using intracoronary or intracardiac infusion of norepinephrine or cAMP to induce VT [19]. Contemporary models focus on other non-ischemic models, for example, in studies of cardiopulmonary resuscitation (CPR). Menegazzi et al. induced VF via transthoracic stimulation with alternating current at 60 Hz, 100 mA, and 3 s of duration [20]. Reynolds and colleagues, on the other hand, induced VF transthoracically using alternating current with a frequency of 603 Hz, an amperage of 1003 mA, and a duration of 3 s [21]. Niemann et al. [22] used a bipolar pacing electrode introduced into the right ventricle to induce VF. After VF induction, animals underwent mono- and biphasic defibrillation with an initial energy of 200 J, followed by 300 J and, if necessary, 360 J. The studies conducted by this group concluded that both monophasic and biphasic defibrillation are effective in terminating VF [22]. Georgiou et al. [23] used a similar VF model obtained by right ventricular stimulation in pigs to investigate the effects of chest and abdominal compression during CPR. Tanaka et al. [24] used a model employing right ventricular stimulation to induce premature ventricular complexes (PVCs) in studies on dilated cardiomyopathy (DCM) induced by PVCs. In this study, the bipolar pacing electrode was placed at the right ventricular apex. De Diego et al. [25] investigated the effects of toxic doses of bupivacaine on myocardial repolarization dispersion and induction of ventricular arrhythmias in pigs, and evaluated the efficacy of lipid emulsion in reversing these effects. Ventricular arrhythmias in this model were induced by intravenous administration of bupivacaine, initially as a bolus at a dose of 4 mg/kg followed by continuous infusion at a dose of 100 μg/kg/min. Ventricular arrhythmias occurred in 43% of the animals, and Brugada-like ECG changes were observed in 36% of the pigs [25]. In another study, the frequency of ventricular arrhythmias during epicardial ablation in pigs and dogs was evaluated [26]. Ventricular arrhythmias were induced during epicardial ablation with a novel partially insulated multipolar epicardial ablation catheter and a Stockert radiofrequency generator. Both unipolar and bipolar ablation were performed for 120 s or until evidence of ablation was seen on the electrocardiogram, depending on which occurred first. The researchers confirmed that the pig model is more susceptible to the development of ventricular arrhythmias during epicardial ablation than the dog model [26]. Vaseghi et al. showed in a chronic porcine post-MI model that vagus nerve stimulation (VNS) reduces VT inducibility by ~60% after ischemia induced via mid-LAD balloon occlusion with microsphere injection; ventricular arrhythmias were induced using programmed ventricular stimulation with two drive cycle lengths and up to three extrastimuli delivered from the right ventricular endocardium and anterior left ventricular epicardium [27]. In another chronic post-MI swine model, Hoang et al. reported that thoracic epidural anesthesia (TEA; C7–T1 lidocaine) decreased PES-induced (programmed electrical stimulation) VT/VF inducibility by ~70% without impairing right ventricular systolic function, consistent with increased ventricular refractoriness/repolarization and a flatter restitution slope [28].

In summary, the domestic pig is an indispensable research model for the study of ventricular arrhythmias. Both ischemic and non-ischemic porcine models provide invaluable information on ventricular arrhythmias. Ischemic models are crucial for understanding arrhythmias associated with myocardial ischemia, highlighting the role of ischemia–reperfusion injury. On the other hand, non-ischemic models are useful for studies of ventricular arrhythmias induced by ablation and cardiac stimulation, as well as those induced pharmacologically. Porcine models remain an important component of cardiovascular research, and their close similarity to human cardiac physiology makes the results of these studies highly relevant for translational research. Summarized characteristics of swine models of ventricular arrhythmias are presented in Table 2.

### 3.2. Sheep Model

One of the advantages of sheep-based models is that their cardiac anatomy is similar to that of humans, including coronary vessels and changes in ventricular muscle after infarction [29]. The main difference lies in the different transmural Purkinje fiber system [30]. This can also be considered a disadvantage of this model.

In the case of sheep, there are well-established models of myocardial infarction, both involving acute ischemia and chronic myocardial infarction, which lead to ventricular remodeling and heart failure [31,32,33]. These models also develop spontaneous ventricular arrhythmias, which are used in ventricular arrhythmia research [33,34]. The aforementioned ventricular arrhythmia models typically rely on inducing spontaneous arrhythmias secondary to myocardial infarction, bradyarrhythmia, or aconitine administration [35]. Most studies are based on inducing myocardial infarction in an ischemic model. This is performed surgically, with opening of the chest cavity and ligation of coronary arteries such as LAD, or less invasively, through temporary occlusion of these vessels using a balloon catheter under fluoroscopic guidance [36,37,38,39,40]. These methods are similar to those used in the porcine model. Thanks to the less invasive method, we can repeatedly and precisely induce ischemia in the desired area, which is very similar to that occurring during myocardial infarction in humans. Reek et al. [36] proposed a very effective model that allows the induction of reproducible monomorphic ventricular tachycardia through programmed stimulation in animals after myocardial infarction. Myocardial infarction was induced by temporary balloon occlusion of the left anterior descending coronary artery. A catheter (8 Fr size, 3 mm balloon inflated to 6 atm) was introduced into the corresponding branch of the left coronary artery, which is the equivalent of the human LAD, for 150 min. VT induction was performed using programmed stimulation from the right and left ventricles, 21 to 43 days after infarction induction. After stabilizing the basic cycle length (350 ms), four additional beats with shortening cycles (280, 270, 260, 250 ms) were induced, and then all intervals were shortened by 10 ms until the desired arrhythmia was obtained. After approximately 3–8 weeks of observation, reproducible ventricular tachycardia lasting from 30 s to 15 min was successfully induced in 80% of the animals [36]. During these studies, no ventricular fibrillation was observed. This model demonstrates a high incidence of inducible sustained monomorphic ventricular tachycardia in the chronic phase of myocardial infarction. Several years later, the author used this method again to attempt mapping of induced ventricular tachycardia [41]. The results of these studies suggest that this inducible ventricular tachycardia results from a macro-reentry mechanism involving subepicardial layers at the border of transmural infarction. Another study concerning the occurrence of ventricular arrhythmias secondary to myocardial infarction was described by Hsieh et al. [40]. It assessed the occurrence and electrophysiological properties of ventricular tachycardias in the early phase after infarction. For this purpose, myocardial infarction was induced in 36 sheep via balloon occlusion of the equivalent of the left anterior descending artery for 3 h. On day 8 and day 100 after infarction induction, electrical mapping of the heart and programmed ventricular stimulation (PVS, pulse width 2 ms, drive train 8 beats, and ≤ 4 premature extrastimuli from the RV apex and basal septum) were performed, among other procedures. The study determined the time of onset and stabilization of VT loops after infarction and the factors that may influence VT inducibility. Ventricular tachycardia induced by PVS was present in 43% of sheep on day 8 after infarction. It was also found that both the distribution of normal and slowed conducting channels of surviving myocytes within the infarction area and increased complex fractionated signals influenced VT induction. Another model described in sheep involves ventricular arrhythmia triggered by bradycardia or a combination of bradycardia and myocardial infarction. The experiment compared the incidence of spontaneous ventricular tachycardia and sudden cardiac death (SCD) in cases of bradycardia alone and in combination with chronic myocardial infarction in sheep [33]. Bradycardia was achieved in all animals through atrioventricular node ablation, and in some animals, balloon occlusion of the left anterior descending coronary artery was additionally performed. The second group of animals showed a significantly higher number of premature ventricular contractions, ventricular tachycardias, ventricular fibrillations, and sudden cardiac death. The combination of myocardial infarction with bradycardia resulting from atrioventricular node ablation in sheep can thus serve as a model for studying VT and sudden cardiac death. In a study on the atrioventricular block model, Farrah et al. demonstrated that sheep are highly susceptible to ventricular arrhythmia during radiofrequency ablation of the His bundle, particularly when the ablation site approached the right ventricle. In the study, non-sustained VT or VF lasting several minutes (cardioverted) was observed in four out of six animals. This information may also be useful in the future for developing models of ventricular arrhythmias. Haissaguerre et al. [42,43,44] demonstrated that arrhythmias originating from papillary muscles can trigger ventricular fibrillation and cause sudden cardiac death even in the absence of structural heart disease.

In the study by Münkler et al. [35], aconitine was administered into the anterior papillary muscle at a mean dose of 0.06 µg (range 0.06–0.15 µg). Aconitine is an arrhythmogenic alkaloid that most likely leads to intracellular accumulation of sodium and calcium [45,46]. The stock solution of aconitine (3 µg/mL) was prepared in 50 mL of distilled water, and one or two drops of 4 M HCl were added to verify solubility. The substance was injected into the papillary muscle under ultrasound guidance, taking particular care to avoid contact between aconitine and the epicardium. The administered doses were gradually increased to induce a stable monomorphic ventricular tachycardia and ultimately ventricular fibrillation. In all animals, focal ventricular arrhythmias were successfully induced, and most of them developed stable ventricular tachycardia [35]. Table 3 shows the characteristics of sheep as animal models of ventricular arrhythmias.

### 3.3. Goat Model

Goats are often used as animal models of arrhythmias, but mainly atrial arrhythmias. The atrial fibrillation model is well established and used to study its mechanisms and to assess the efficacy and potential proarrhythmic effects of various antiarrhythmic drugs [47]. Due to the relatively constant anatomy of the coronary vessels, goats can also be used in studies on myocardial infarction [48]. However, it should be noted that goats are not commonly used as models of ventricular arrhythmias.

### 3.4. Horse Natural Model

Horses are natural athletes that have evolved over many years to develop the athletic abilities necessary to avoid predators and are a model for ventricular arrhythmias in athletes. Horses participate in athletic activities that imitate human activities, ranging from occasional/recreational exercise to daily/intense conditioning. Sudden cardiac death is a leading cause of death in human athletes and is 2.8 times more likely to occur in athletes than in non-athletes [49]. Horses may be a particularly good model to investigate the function of the potassium voltage-gated channel Kv1.5 due to its spontaneous ventricular expression, which is lacking in human ventricles [50]. However, available studies usually address the clinical problem of ventricular arrhythmias in horses without treating the horse strictly as a model animal [51]. Much more often, horses, like goats, are a model of atrial fibrillation [52].

### 3.5. Canine Model

#### 3.5.1. Ischemic Ventricular Arrhythmia Models

For studying the pathological ischemia-driven mechanisms of ventricular arrhythmias, dogs serve as excellent models, as their hearts are structurally, electrophysiologically, and even behaviorally similar to those of humans in relation to ischemic damage. Dogs exhibit arrhythmias during ischemia due to both reentry and ectopic automaticity, which is typical in human patients. Compared to smaller models like mice or rabbits, dogs present a more well-rounded and integrated picture of complex ischemic responses. The studies suggest that dogs, like humans, exhibit a biphasic framework of ischemic ventricular arrhythmias and are therefore considered to have greater translational relevance than rodents, which exhibit only a single phase [53,54]. A biphasic action potential indicates human repolarization better and a greater chance of succumbing to arrhythmias. The vast reach of the Purkinje fibers in dogs’ hearts triggers reentry circuits, which are the most important substrate for ventricular fibrillation. They exhibit heart rates from 60 to 120 bpm, which are closer to the actual heart rates of humans, making them better at modeling the arrhythmia-inducing mechanisms and anti-arrhythmic drug actions as opposed to rodents, whose heart rate is 5 to 10 times higher. Furthermore, dogs have well-established collateral circulation, which is beneficial for studying ischemic preconditioning and evaluating therapeutic interventions. Various animal species, such as the domestic dog, offer a good rationale for studying ischemic preconditioning or evaluating therapeutic treatments. Perhaps the most important aspect is that they also have very good autonomic control, with significant sympathetic and parasympathetic control of conduction and excitability. The strong sympathetic nervous system activity, which is one of the important reasons for arrhythmias in humans, can be induced in dogs, allowing the study of baroreceptor function and autonomic modulation treatments.

At the mechanistic level, increasing evidence indicates that the autonomic contribution to ischemia-induced ventricular arrhythmias in canine models is mediated by complex interactions within the intrinsic and extracardiac cardiac nervous system. Experimental studies have demonstrated that intrathoracic extracardiac neurons, particularly those located within the middle cervical ganglion, actively transduce regional ventricular ischemia and contribute to reflex sympathetic excitation, thereby promoting ventricular electrical instability. Importantly, neuromodulation using dorsal spinal cord stimulation selectively attenuates neural responses to regional myocardial ischemia without suppressing responses to global cardiac stress, highlighting a mechanism by which ventricular arrhythmogenesis may be mitigated without impairing physiological cardiac control [55]. Complementary network-level investigations of the intrinsic cardiac nervous system further revealed that local circuit neurons integrate ischemic afferent signals with central sympathetic and parasympathetic inputs through dynamic, stochastic interactions, and that dysregulation of these networks may facilitate arrhythmia initiation. Together, these findings underscore the critical role of cardiac neural control in modulating ischemia-related ventricular arrhythmias and provide a mechanistic framework supporting the translational relevance of canine models for studying neuromodulatory therapeutic strategies [56].

##### Experimental Ischemic Models

Most animal models used to study ischemia-induced arrhythmias use either Harris’ 2-stage model or a variant. Many studies have examined hemodynamics and arrhythmias following ligation of one or more coronary arteries [57]. The Harris method requires two sutures to be placed around the left anterior descending coronary artery. One of the sutures is tied tightly around a 20-gauge hypodermic needle, which is immediately removed, leaving stenosis but not occlusion of the coronary artery. Thirty minutes after, a second ligature is tightly applied, completely occluding the coronary artery. Probably the greatest effect on animal survival, despite the two-stage ligation of the main coronary artery, is due to preconditioning, in which the integrity of the K_ATP_ channels is maintained and the outflow of potassium ions from the cell is reduced [58,59,60].

The model devised by Janse and Wit [57] carries out a thorough electrophysiological approach to ischemia-related ventricular arrhythmias. The analysis emphasizes acute ischemia and reperfusion with respect to transmembrane potential and conductivity across the myocardium, bearing in mind the myocardial layers. In this case, regional coronary occlusion was produced in anesthetized dogs to follow the progression of ischemic electrophysiologic phenomena. Microelectrode recordings enabled measurement of action potentials in the ventricular myocardium and Purkinje fibers, which illustrates the contribution of depolarization heterogeneity to arrhythmia. The work showed that after acute ischemia, the active phase of Purkinje fibers is longer than that of the adjacent myocardial cells, so the conditions for reentry and ventricular tachycardia are present. Furthermore, ischemic areas demonstrated a progressive decrease in the resting membrane potential, leading to prolongation of the refractory period and slowing of conduction, all of which is conducive to malignant ventricular arrhythmias. The work also made important contributions to understanding reperfusion arrhythmias, where the sudden restoration of blood flow is shown to be capable of provoking ectopic activity and increasing the vulnerability of the heart to ventricular fibrillation. The in vivo nature of the model enabled continuous, real-time arrhythmia monitoring, which closely simulates the clinical setting of myocardial infarction and revascularization. The Janse model in canines enhances previous methods by combining precise electrophysiological mapping with dynamic ischemia–reperfusion processes. This model serves to understand the mechanisms of ventricular tachycardia and fibrillation in the setting of acute and subacute ischemia, analyze the involvement of Purkinje fibers in arrhythmogenic processes, and assess the effectiveness of antiarrhythmic treatment aimed at conduction disturbances of the heart.

The Folt modification of the Harris Technique, described in 1991 [61], provides a robust methodology for the study of sub-acute myocardial infarction and thrombogenesis in the context of human pathology. In this procedure, a loose ligature is placed on a major coronary artery, with a probe positioned downstream of the ligature that is attached to a blood vessel. The ligature is then tightened to reduce arterial flow by more than 50%, and the narrowed segment is repeatedly compressed three to six times using a hemostat or similar instrument. Alternatively, a small wire can be inserted into the same or another coronary artery; when an electrical current is applied, a clot forms, as demonstrated by Minami et al. [62]. Both techniques initiate a clotting cascade, leading to the release of clots into the myocardial region. After about two hours, the procedure leads to some pathological myocardial infarction in addition to the ectopic impulses, but the scope of obstruction, along with ischemia, permutation is relatively greater than in the original Harris technique.

The Harris method, initially described by Sabbat et al. [63], involves the stepwise injection of approximately 100 µm diameter polystyrene microspheres into a major coronary artery. No more than 10 injections can be done in total, and each can be done 1 to 3 weeks apart. Throughout the process, left ventricular shortening fraction is monitored through echocardiography, which makes it possible to observe gradual dysfunction of the heart and the development of heart failure. Transient ventricular arrhythmias usually follow each injection, and while they do tend to subside, a rest period is prescribed following the last injection; when complete heart failure presents, ventricular ectopy may return. One of the main benefits of this technique is its independence from collateral circulation because, after the microsphere partially obstructs small arterial branches, it serves to impede the development of compensatory collateral vessels. This model accurately replicates the gradual loss of functional myocardial tissue, with the final stage representing stable and reproducible chronic heart failure.

In 1980, Schwartz and Stone [64] developed a canine model with a prior myocardial infarction (MI), ischemia, and exercise-induced sympathetic overactivity. The purpose of the study was to evaluate the possible impact of left-sided sternotomy (LSGx) on the incidence of ventricular fibrillation induced by acute myocardial ischemia. The experiment was conducted on 32 mongrel dogs. After inducing anterior myocardial infarction, they were randomly assigned to either the control or the left-sided stellectomy (LSGx) group. After three weeks, all the dogs underwent controllable occlusion of the left circumflex artery to produce acute ischemia. The control group suffered from VF in 65% of the subjects, while the LSGx group only yielded 33%. Mortality also differed significantly between groups [64]. The study concluded that left-sided stellate artery ligation significantly reduced the incidence of VF in dogs subjected to acute ischemia after myocardial infarction.

The model developed by Issa et al. [65] is somewhat similar: dogs have a healed MI and superimposed acute ischemia, but in the presence of pacing-induced heart failure rather than exercise, and the model does not require a thoracotomy. The anesthesia used in the study is the most significant difference from the Schwartz model. This study aimed to create and assess a novel non-thoracotomy animal model to study the pathophysiological mechanisms of sympathetic-induced ventricular arrhythmias in the context of healed MI, heart failure, and increased sympathetic tone. The study consisted of two surgeries separated by a 5-week recovery period. The first stage involved pacemaker implantation and induction of an MI. After 2 weeks of recovery, heart failure was achieved by rapid ventricular pacing at a rate of 200 to 240 bpm for 3 weeks. Rapid pacing was stopped just prior to the second surgical procedure. A coronary balloon dilation catheter was placed over the angiographic catheter into the proximal part of the circumflex artery of the left coronary artery (LCX) just before the first marginal branch. The balloon was then inflated to completely occlude the proximal LCX for 4 min, effectively inducing acute myocardial ischemia. VT/VF developed in 72% of the animals [65]. This canine model serves as a valuable tool for investigating ventricular arrhythmias in a setting that closely replicates clinical conditions, where multiple factors, including a healed myocardial infarction, heart failure, and heightened sympathetic activity, contribute to the development of acute myocardial ischemia. Similar to other models, it provides an opportunity to assess innovative prophylactic antiarrhythmic strategies.

The canine model described by Hirahara et al. in 2024 [66] improves upon previous ischemia-based arrhythmia models by implementing controlled ischemia–reperfusion sequences for studying VT induction. In this model, full reperfusion is administered after the left anterior descending coronary artery has been occluded for 120 min, mimicking clinical situations like percutaneous coronary intervention. A few days later, programmed electrical stimulation is applied to elicit VT, providing a reproducible means to evaluate ischemia-induced arrhythmias. In this particular study, 80% of the dogs had VT successfully induced, with a mean heart rate of 335 ± 70 bpm. Episodes frequently exhibited differing QRS morphologies, consistent with the variability observed in clinical cases [66]. Unlike other models that use permanent coronary artery occlusion, this methodology includes reperfusion, thereby bringing the simulation closer to the reality of post-revascularization events of arrhythmia. Moreover, it allows reproducible VT induction in a single subject, which is helpful for studying antiarrhythmic treatment, defibrillation, autonomic modulation, and other interventions during acute myocardial ischemia and reperfusion.

Canine models are essential for studying ventricular arrhythmias, and their specific contributions to understanding ischemia-related arrhythmogenic phenomena are extensive. While older models like the Harris method use controlled coronary ligation, capturing the effects of ischemia on repolarization and arrhythmia development, modifications like Folts and Sabbah introduce additional elements like thrombogenesis and progressive myocardial dysfunction, allowing for the study of clot formation, collateral circulation, and chronic heart failure-related arrhythmias. Schwartz and Issa’s models add heart failure and sympathetic modulation along with the previously mentioned heart failure and provide a more realistic setting to evaluate antiarrhythmic treatment effectiveness. The model by Hirahara et al., on the other hand, focuses on the post-hypoxic phase of ischemia and its arrhythmic aftermath during the revascularization process, and so draws attention to the back-and-forth shifting between ischemia and reperfusion states. Each of these models serves a distinct purpose in advancing our understanding of ventricular arrhythmias and offers a tailored platform for evaluating therapeutic strategies in settings that closely mimic clinical conditions.

#### 3.5.2. Experimental Non-Ischemic Models

##### Epinephrine-Induced Arrhythmias

The studies by Todd and Vick (1971) [67] and Brock (2003) [68] provided a great deal of detail concerning the mechanisms of arrhythmias associated with the action of epinephrine using in vivo canine models, which allowed for the analysis of the effects of adrenergic stimulation on the heart under physiological conditions.

The Todd and Vick model analyzed the action of epinephrine on potassium concentration in plasma and the roles of adrenergic receptors in ventricular arrhythmias [67]. The study was performed on dogs that received epinephrine injections, during which their cardiac electrophysiology, blood pressure, and plasma ion concentrations were measured. The findings demonstrated that higher levels of adrenergic activity produce pronounced electrolyte derangements, particularly hypokalaemia, which predisposes to the development of ventricular arrhythmias. This means that powerful adrenergic stimulation can cause the myocardium to be overly excitable, particularly during stressful situations or in excessive catecholaminemia.

In Brock’s model, in addition to the influence of epinephrine, the sensitivity of the heart to arrhythmias under conditions of exposure to volatile organic compounds was also analyzed [68]. The results showed that the combination of epinephrine and exposure to environmental toxins (hydrofluorocarbons) can significantly increase the tendency toward ventricular arrhythmia, suggesting that epinephrine’s effect on the heart is further modulated by environmental factors. Brock’s model, unlike the Todd and Vick model, which concentrated on electrolyte interactions and the stimulation of adrenergic receptors, incorporated an additional environmental factor that contributes to the development of arrhythmia, which increases the complexity of the model for the purposes of broader human clinical medicine.

The intricate details of the complex mechanisms that lead to ventricular arrhythmias due to substantial adrenergic excitation are revealed and systematized in the two models. The canine model is of great translational value, as it is known that the dog’s autonomic nervous system and its response to catecholamines bear resemblance to those in humans, aiding in the understanding of neurogenic control of heart rate and the possible management of adrenergic arrhythmias.

##### Torsade de Pointes

The goal of the Bardy et al. study was to analyze the sequence of epicardial activations in relation to torsade de pointes (TdP) in dogs [69]. To induce the arrhythmia, 30 mg/kg of quinidine was administered, resulting in QT interval prolongation and the induction of early afterdepolarizations (EADs). In addition, occlusion of the left anterior descending artery (LAD) was performed in some dogs to assess the effect of ischemia on TdP mechanisms. The study used cardiopulmonary bypass, which helped maintain hemodynamic stability, providing a better perspective on the electrophysiological mechanisms involved. Analysis of epicardial activation showed that TdP is characterized by dynamic changes in the depolarization sequence, which suggests that the arrhythmia results from competition between different foci of activation [69]. This study complemented the understanding of TdP development, while combining prolonged QT with ischemia, and helped understand the spatial variation in ventricular activation in this arrhythmia.

The study done by Inoue et al. [70] evaluated TdP in dogs where the QT interval was also prolonged due to a high dosage of quinidine (30 mg/kg). Unlike in the Bardy model, this experiment was done on dogs with no prior myocardial infarction, enabling the assessment of TdP in the setting of prolonged QT in isolation from ischemic influences. Programmed ventricular stimulation was used to induce arrhythmia, and epicardial activation mapping was performed. The findings indicated that, in this model, the arrhythmia was not due to competing activation sequences but rather to a shift in the most anterior epicardial activation region, suggesting a mechanism dependent on repolarization heterogeneity [70]. This study confirmed that TdP can result from dynamic changes in the location of ectopic beats, rather than from typical reentry mechanisms.

The model described by Vos et al. [71] was on dogs having chronic atrioventricular block bradycardia, which was associated with QT prolongation. The d-sotalol—a class III potassium channel blocker and a drug used for treating bradycardia—was used in the experiment. Programmed cardiac stimulation was used to cause TdP, analyzing different stimulation patterns, one of which was ‘short-long-short’ protocol (two short coupling intervals separated by one longer interval), which was found to be the most effective in evolving arrhythmia [72]. The findings from the study indicate that in this model, the occurrence of TdP was predominantly linked to the presence of EAD along with regional repolarization heterogeneity. Moreover, the study demonstrated that TdP can be prevented by administering magnesium sulfate (MgSO_4_) or isoprenaline, which is of remarkable clinical importance for antiarrhythmic treatment [71].

The research conducted by El-Sherif et al. developed a model of TdP, which was induced using the toxin AP-A [73]. This specific neurotoxin caused QT prolongation and bradycardia due to disruption of the nervous system. The study employed three-dimensional electrophysiological mapping, enabling accurate investigation of repolarization mechanisms and arrhythmia-inducing foci. The study showed that TdP developed due to an abrupt change in the length of the cardiac cycle, resulting in the phenomenon being associated with spatial heterogeneity of repolarization. This model was significant in providing information about the influence of TdP on autonomous heart regulation and possible mechanisms that lead to the spontaneous arrhythmia in people with congenital and acquired long QT syndrome.

A study by Nayebpour et al. investigated the effect of cesium chloride on the electrophysiology of the dog heart [74]. The substance was known to block potassium current, which makes the QT interval longer, but its effects were less consistent when compared with aggressive drugs like sotalol or quinidine. In the experiment, cesium chloride infusion led to numerous ventricular tachyarrhythmias; however, these rarely took the classical form of TdP. A transition to ventricular fibrillation was observed more frequently, which suggests that cesium chloride may not be the optimal tool for modelling TdP. This study reinforced the idea that mechanisms of TdP are not solely reliant on prolonged QT intervals, but also on how a particular chemical modifies ionic repolarization mechanisms.

Each of the presented models contained relevant information about the mechanisms of TdP. The model of Vos et al. turned out to be one of the most reproducible and clinically translatable, whereas El-Sherif et al. brought forth novel information regarding heart autonomic regulation and its influence on the progression of TdP. The model of Nayebpour et al. showed limitations in using cesium chloride for studying this arrhythmia, while the studies by Bardy and Inoue revealed differences in the mechanisms of TdP depending on the presence of ischemia and the type of antiarrhythmic drugs used.

#### 3.5.3. Natural Models of Ventricular Arrhythmias

##### German Shepherd Model of Sudden Death

Ventricular arrhythmias, specifically those observed in German Shepherd Dogs (GSDs), present an unusual model for studying inherited heart conditions that lead to sudden death. These arrhythmias, which first manifest in early adolescence, are typically associated with specific genetic traits in this breed. It was noted that frequent premature ventricular complexes (PVCs) and ventricular tachycardia (VT) tend to be associated with sudden cardiac death, typically occurring between 4 and 18 months of age [75].

One of the hallmark features of these kinds of GSD arrhythmias is their age dependence. Young dogs under 12 weeks of age generally have few, if any, visible arrhythmias. However, as they age, the frequency and severity of arrhythmic events significantly increase. Dogs between 22 and 26 weeks of age start to display significant arrhythmias, such as frequent PVCs and polymorphic VTs [76]. This observable phenomenon indicates that these arrhythmias are modulated by cumulative changes, especially developmental processes related to heart rate-regulating mechanisms. It has been noted that arrhythmias tend to be prominent during the so-called sinus bradycardia hypoactivity or during the pauses, which are either spontaneous or drug-induced [75,76].

The pathophysiology of these arrhythmias involves abnormalities in ventricular myocardial cells, particularly in the balance between sympathetic and parasympathetic nervous system activity. Research on the sympathetic innervation of the heart indicates that GSDs with inherited arrhythmias might demonstrate some form of abnormal sympathetic innervation, especially in the anteroseptal left ventricle [77]. This abnormal innervation leads to an enhanced response to β-adrenergic stimulation, which is likely to cause delayed afterdepolarizations (DADs) and other forms of arrhythmic activity [77].

Electrophysiological studies have revealed that these dogs also exhibit significant differences in their cardiac electrophysiological properties compared to unaffected dogs. For example, when challenged with drugs like isoproterenol, GSDs with arrhythmias show more pronounced changes in action potential duration and a higher incidence of triggered activity [77]. This triggered activity is of particular concern because it initiates arrhythmias such as VT. Moreover, studies have shown that these dogs have more arrhythmias under more specific conditions, such as the presence of stressors, including β-adrenergic agonists, and during bradycardia [75].

The genetic basis for these arrhythmias remains under investigation, though it is clear that the condition is inherited. Pedigree analyses of affected GSDs have shown that the disorder likely follows an autosomal dominant pattern of inheritance, with significant variation in symptom severity among affected dogs [78].

GSDs with inherited ventricular arrhythmias provide a valuable natural model for studying the mechanisms underlying sudden cardiac death, particularly the role of age, heart rate variability, and sympathetic nervous system dysfunction in arrhythmia initiation. Further research into these mechanisms may offer insights into similar conditions in humans, especially those that lead to sudden death in apparently healthy individuals.

##### Boxer Model of Sudden Death

Pedigree analysis of affected boxers has revealed that their ventricular arrhythmias are inherited as an autosomal dominant trait [79]. Sudden death in affected boxers commonly occurs in adulthood, with a broad age range of 2 to 8 years. While sudden death may be the first clinical sign, most boxers experience episodes of syncope. Both death and syncope are often associated with exercise and excitement [80]. Although some dogs have coexisting myocardial failure, most with VT show no clinical signs of congestive heart failure and have normal echocardiographic results. The ventricular arrhythmia may be merely rare ventricular premature depolarizations with or without fixed coupling, paroxysms of ventricular tachycardia, or sustained tachycardia. The electrocardiographic and histologic features that characterize boxers with sudden death are similar to arrhythmogenic right ventricular dysplasia. Therefore, they can be used as a model for this disease. 

The natural model of arrhythmogenic right ventricular cardiomyopathy (ARVC) in boxer dogs, described by Basso et al. [81], is among the most thoroughly characterized spontaneous animal models of this disease and shows remarkable similarity to its human counterpart. The study analyzed 23 dogs presenting with sudden death, syncope, and ventricular arrhythmias of suspected right ventricular origin. Each animal underwent a comprehensive diagnostic evaluation, including 24 h Holter monitoring, full necropsy, and quantitative histopathological and immunohistochemical assessment (H&E, Heidenhain trichrome, CD45/CD43/CD68 markers), as well as apoptosis detection using the TUNEL assay. In selected cases, MRI of fixed tissue samples was performed, allowing precise correlation between regions of high signal intensity and histologically confirmed adipose infiltration.

Across all examined hearts, investigators identified extensive transmural loss of right ventricular cardiomyocytes with replacement by adipose or fibrofatty tissue, predominantly affecting the infundibular and anterolateral regions. These lesions formed islands of surviving myocardium embedded within adipose tissue, a hallmark morphological pattern of ARVC. Additionally, 61% of dogs exhibited myocarditis, and nearly 40% showed evidence of cardiomyocyte apoptosis, indicating that inflammatory–degenerative processes likely accelerate myocardial injury and create a highly arrhythmogenic substrate. Clinically, many dogs demonstrated episodes of ventricular tachycardia and very frequent premature ventricular complexes, while sudden death often occurred as the first manifestation. Familial clustering was observed in several cases, supporting an autosomal dominant mode of inheritance consistent with human ARVC.

The integration of advanced histologic, immunohistochemical, and imaging techniques with a clear and clinically significant arrhythmogenic phenotype makes the boxer model described by Basso et al. a highly valuable translational tool. It enables detailed investigation of mechanisms underlying sudden cardiac death—including fibrofatty remodeling, myocardial inflammation, apoptosis, and conduction abnormalities—and provides a robust platform for evaluating emerging diagnostic strategies and therapeutic interventions relevant to human cardiovascular medicine. They documented a high degree of clinical and pathological similarity to this disease in humans.

Oyama et al. [82] performed a comprehensive analysis of calcium-handling abnormalities in boxer dogs with ARVC, integrating molecular, biochemical, and electrophysiological approaches. Methods included oligonucleotide microarray profiling, RT-qPCR validation, RyR2 immunoprecipitation, immunoblot quantification of calstabin-2 (FKBP12.6), and single-channel recordings using planar lipid bilayers to assess RyR2 gating behavior. Affected dogs displayed markedly reduced calstabin-2 levels in the RyR2 complex despite normal RyR2 expression, indicating selective disruption of channel stabilization. Single-channel analyses revealed increased open probability of RyR2, consistent with pathological diastolic Ca^2+^ leak—a well-established mechanism leading to ventricular tachyarrhythmias. This model provides one of the few large-animal, naturally occurring examples of Ca^2+^-leak-mediated ventricular arrhythmias, offering exceptional translational value for studying ion-handling defects relevant to human ARVC and catecholaminergic arrhythmia syndromes.

Meurs et al. [83] conducted a detailed genetic investigation in boxer dogs with ARVC and identified an 8 bp deletion in the 3′UTR of the *striatin* (STRN) gene, strongly associated with the disease phenotype. The study included targeted PCR, direct DNA sequencing of STRN regions, and mRNA expression analysis, combined with phenotypic assessment based on 24 h Holter monitoring. Dogs homozygous for the mutation exhibited markedly higher premature ventricular complex (PVC) counts. Immunofluorescence localization studies demonstrated that *striatin* colocalizes with key desmosomal proteins within the intercalated disc, reinforcing its role in mechanical coupling and arrhythmogenesis. This work established a spontaneous, genetically defined large-animal model of desmosomal disease, providing a valuable parallel to human ARVC and enhancing the translational relevance of boxer dogs in ventricular arrhythmia research.

Oxford et al. [84] used quantitative transmission electron microscopy (TEM) to evaluate intercalated disc structures in eight boxer dogs with histopathologically confirmed ARVC. The methodology involved precise measurement of desmosomes, gap junctions, and adherens junctions in both right and left ventricles, using standardized tissue processing protocols and ImageJ-based morphometric analysis (Image J 1.38X software). The study demonstrated significant reductions in the number of these junctional complexes, as well as shortened desmosomal lengths, reflecting impaired structural coupling. Additionally, the authors observed electron-dense material originating from the Z-band and extending into the sarcomere, along with cytoskeletal disorganization assessed via α-actinin immunoblotting. These ultrastructural abnormalities closely mirror those reported in human ARVC and constitute a clear anatomic substrate for ventricular arrhythmias, supporting the boxer as a robust translational model for studying impaired cellular connectivity and arrhythmogenic remodeling.

##### Doberman Pinscher Dilated Cardiomyopathy

Approaching the Doberman pinscher as a natural model for studying functional ventricular arrhythmias in dilated cardiomyopathy (DCM) provides fundamental insights into the role of these dogs in arrhythmia research and cardiac arrest. Dobermans have an inherited disposition to develop DCM. The hallmark of this disease is the presence of ventricular arrhythmias like premature ventricular complexes and episodes of ventricular tachycardia [85]. These arrhythmias often progress to more severe forms, including ventricular fibrillation, which can result in sudden death. The natural progression of DCM in Dobermans is well established, with the disease typically following a three-stage progression. The first stage is the occult stage, where the patient exhibits no clinical signs, but electrical events such as PVCs can be detected. The next stage is associated with heart failure. In the end, the dog may die suddenly or along with congestive heart failure. For this reason, Dobermans may serve as excellent research animals for the study of arrhythmia pathogenesis and mechanisms of death.

Calvert et al. [86] described a large group of Doberman pinschers (*n* = 54) with occult dilated cardiomyopathy (DCM), demonstrating that these dogs frequently develop premature ventricular complexes (PVCs), non-sustained and sustained ventricular tachycardia (VT), and sudden cardiac death. The study employed comprehensive echocardiographic evaluation, 24–48 h Holter monitoring, and detailed necropsy with histopathological analysis of dogs that died suddenly. Importantly, malignant ventricular arrhythmias occurred even in the occult phase, when echocardiographic parameters were still within normal limits. Histology consistently revealed diffuse myocardial fibrosis, myocyte degeneration, and fatty replacement—structural abnormalities that closely mirror the arrhythmogenic substrate of human DCM. This work positions the Doberman as a robust translational model for studying early ventricular arrhythmogenesis, risk stratification, and therapeutic responses in dilated cardiomyopathy.

Smucker et al. [87] were the first to propose the Doberman pinscher as a natural, large-animal analogue of human dilated cardiomyopathy with a high risk of ventricular arrhythmias. In a controlled comparison of Dobermans (n = 30) and mixed-breed dogs (*n* = 41), the authors combined two-dimensional echocardiography, cardiac catheterization, and coronary blood flow measurements using microsphere techniques. Dobermans showed significantly reduced left ventricular fractional shortening, chamber dilation, and impaired contractility despite minimal clinical signs—changes that represent the functional underpinnings of human DCM. These structural and systolic abnormalities create a substrate highly susceptible to ventricular ectopy and tachyarrhythmias. The study laid the groundwork for using the Doberman as a translational model to investigate the progression from early LV remodeling to clinically significant ventricular arrhythmias and sudden cardiac death.

Wess et al. [88] identified a subset of Doberman pinschers with DCM in which disease progression and ventricular arrhythmias were associated with circulating functional β1-adrenergic receptor autoantibodies (β1-AAB). In a group of 118 dogs, the researchers used a cardiomyocyte bioassay to quantify β1-AAB activity and correlated these findings with Holter-derived ventricular arrhythmia burden and echocardiographic indices. Dogs with high β1-AAB activity exhibited significantly more PVCs, increased incidence of VT, and reduced survival. Notably, therapeutic agents known to neutralize human β1-AAB (including loop-mimicking peptides and aptamers) inhibited the activity of the canine antibodies in vitro, highlighting a direct mechanistic parallel to autoimmune DCM in humans. This model provides a unique translational platform for studying immune-mediated ventricular arrhythmias and for developing targeted immunomodulatory therapies relevant to human cardiology.

The relevance of Dobermans as a model extends to their usefulness in evaluating therapeutic strategies to control ventricular arrhythmias and modify the natural course of dilated cardiomyopathy. In the comprehensive review by O’Grady and O’Sullivan [85], these dogs were highlighted as a particularly valuable population for assessing the efficacy of antiarrhythmic interventions, as their disease progression closely mirrors that seen in human DCM. Clinical studies have demonstrated that therapies such as β-blockers and ACE inhibitors can reduce ventricular ectopy, improve hemodynamic stability, and delay the onset of congestive heart failure. Moreover, the predictable progression of the disease—from an occult arrhythmogenic phase to overt myocardial dysfunction—combined with a high incidence of malignant ventricular tachyarrhythmias and sudden death, allows for systematic evaluation of prophylactic and disease-modifying treatments. Due to the heritable nature of DCM in this breed, Dobermans also provide a consistent and repeatable model for investigating long-term therapeutic responses and preventive strategies. Collectively, these features make the Doberman an invaluable large-animal model for translational studies aimed at improving therapeutic approaches for ventricular arrhythmias and heart failure in humans.

The main experimental and naturally occurring canine models of ventricular arrhythmias discussed above are summarized in Table 4.

### 3.6. Feline Model

#### 3.6.1. Ischemic Ventricular Arrhythmias Models

For studying the pathological ischemia-driven mechanisms of ventricular arrhythmias, cats, like dogs, serve as valuable models due to their structural and electrophysiological similarities to human hearts. Nevertheless, cats differ from dogs and humans in that they have a more monophasic action potential, which means there is less difference between layers of myocardial repolarization, rendering the feline heart less prone to re-entry-type arrhythmias. While cats do not exhibit the biphasic type of ischemic ventricular arrhythmias, their neurogenic control of cardiac function makes them ideal subjects for examining the autonomic contribution to arrhythmogenesis. Compared to rodents, cats possess a more developed autonomic balance with well-established sympathetic and parasympathetic control, which is important in ischemic arrhythmias. Their heart rate, although higher than that of humans, remains within a range that allows research on ischemic conduction disturbances. In addition, evidence suggests that, like dogs, cats have a significant neural component in ischemic arrhythmias, making them particularly useful for studying the autonomic system, baroreceptors, and neurogenic modulation of arrhythmogenicity.

##### Experimental Ischemic Model

The model described by Gillis in 1971 provided significant evidence on the neurogenic modulation of ischemia-induced ventricular arrhythmias using a feline model [89]. The issue poses a problem with the autonomic nervous system brought out in an animal model where the left coronary artery was occluded, and sympathetic and/or vagal activities were monitored. Cats were put under anesthesia and underwent neurosurgical procedures like spinal cord C-1 level section and bilateral vagotomy, which offered the possibility of examining the central and peripheral control of the heart. The findings established that there was a rapid increase in both sympathetic and vagal nerve activity following ventricular tachycardia and fibrillation after coronary artery occlusion. In animals where the autonomic nervous system influence was cut off, mortality from arrhythmia was very low with long survival time after ischemia. This study established that ventricular arrhythmias persisted even in the absence of local myocardial factors responsible for autonomic changes, suggesting that these changes are reflex in origin and reflect increases in ischemic arrhythmogenicity. This model was especially useful for examining the relationship between the heart and the autonomic nervous system, providing valuable insights into the neurogenic sources of arrhythmias and ways to treat them.

#### 3.6.2. Experimental Non-Ischemic Models

##### Digitalis Arrhythmias

The study by Somberg employed a feline model to study the autonomic nervous system’s contribution to the mechanisms of digitalis glycoside-induced ventricular arrhythmias [90]. In this experiment, the sodium-potassium pump inhibitor, ouabain, was given as an injection, which resulted in increased levels of intracellular calcium in cardiomyocytes and increased excitability of the myocardium. The study concluded that digitalis arrhythmias have not only a cardiac origin but also a strong neurogenic origin. Transection of the spinal cord at the C-1 level markedly increased the digitalis dose required to produce toxicity, suggesting some degree of central nervous system control over arrhythmia development. Vagal stimulation of the postrema area induced ventricular tachycardia, demonstrating that the nervous system can also depress cardiac excitability induced by digitalis. The use of metoclopramide, a dopamine receptor blocker, increases the threshold of arrhythmia induced by ouabain, which shows how peripheral and central neurogenic mechanisms of digitalis’ arrhythmias can be modulated pharmacologically. The cat heart model has aided in understanding the relationship between the heart and the nervous system during digitalis poisoning and suggests potential treatment approaches through neurogenic modulation. This model cannot ethically be used in living intact animals because the use of digitalis glycosides causes intense and uncontrollable vomiting.

##### Epinephrine-Induced Arrhythmias

The research performed by Muir et al. using cats gave insight into anesthetic interactions with epinephrine-induced ventricular arrhythmias [91]. The study examined the impact of halothane, ether, and cyclopropane anesthetics on the heart rate and on the propensity for conduction disturbances during periods of heightened adrenergic activity. The cats underwent anesthesia with halothane at concentrations ranging from 1 to 3% blended with nitrous oxide and oxygen. They were then monitored for changes in heart rhythm by assessing their hemodynamic and electrophysiological parameters. In conjunction with halothane, sympathetic stimulation induced an arrhythmogenic effect that increased the propensity to ventricular arrhythmias, particularly in the case where epinephrine was administered. Most cats showed multiple ectopic beats and interference dissociation 15 min after the start of anesthesia, and this effect was further enhanced after intravenous epinephrine. Such disorders were not seen in ether anaesthetized cats, leading to the conclusion that halothane does, in fact, increase cardiac sensitivity to adrenergic stimulation. In cases of hyperventilation, the arrhythmias were eliminated only under low carbon dioxide conditions, whereas an elevated carbon dioxide concentration resulted in persistent arrhythmias; this suggests that hypercapnia plays an important role in the genesis of these arrhythmias, likely through respiratory acidosis-mediated alterations in myocardial electrophysiological properties. Specifically, the decrease in pH affects ion channel function and alters action potential duration, which can promote re-entrant circuits and triggered activity [92]. Furthermore, hypercapnia has been shown to increase circulating catecholamines and significantly lower the arrhythmogenic dose of epinephrine (ADE), especially under halothane anesthesia, which further sensitizes the myocardium to adrenergic stimulation [93].

#### 3.6.3. Natural Feline Model of Arrhythmogenic Right Ventricular Cardiomyopathy (ARVC)

Fox et al. [94] presented a group of 12 domestic cats with naturally occurring arrhythmogenic right ventricular cardiomyopathy (ARVC), providing one of the few non-canine spontaneous models of ventricular arrhythmias with strong translational relevance. Using ECG, echocardiography, gross pathology, and detailed histopathology, the authors demonstrated that affected cats exhibited frequent ventricular ectopy, including PVCs and polymorphic ventricular tachycardia, while 5 of 8 monitored animals showed right bundle branch block, closely mirroring the electrophysiologic phenotype of human ARVC. Morphological analysis revealed right-ventricular enlargement in 2.7/3 severity score vs. 0 in controls (*p* < 0.001) and right-atrial dilation (2.8 vs. 0; *p* < 0.001), while left-sided chambers were largely unaffected—reproducing the chamber specificity characteristic of the human disease. Histopathology showed classic fibrofatty myocardial replacement in 9/12 cats, pure fatty infiltration in 3/12, and apical or free-wall right-ventricular aneurysms in 6/12, all of which are hallmark features of human ARVC. Immunohistochemistry demonstrated extensive apoptosis (TUNEL-positive) with indices reaching up to 28% of myocytes in severely affected RV regions, along with inflammatory infiltrates in 10/12 cats, highlighting mechanisms strongly implicated in human ARVC.

Although this model closely recapitulates the structural and electrophysiologic substrate of human ARVC, the authors noted several limitations: the small sample size, absence of identified genetic mutations, and the tendency of affected cats to die from progressive right-sided heart failure rather than sudden death, although a similar congestive phenotype is observed in a subset of human ARVC patients. Despite these limitations, spontaneous feline ARVC remains a uniquely valuable translational model for studying ventricular arrhythmias, fibrofatty myocardial remodeling, inflammation, and apoptosis-driven arrhythmogenesis in a naturally occurring large-animal context. Feline models used in research on ventricular arrhythmias are presented in Table 5.

## 4. Conclusions

Life-threatening ventricular arrhythmias remain a serious medical problem. Despite significant progress in research into their causes and prevention, the mechanisms of ventricular arrhythmias associated with myocardial infarction, ischemia, and drug-induced arrhythmias are still not fully understood. In vivo animal models play an important role in these analyses because they partially reflect human conditions, although the results must be interpreted with caution due to interspecies differences. At the same time, research methods that limit the use of animals are being sought due to ethical concerns. A stable and reproducible model for evaluating ventricular arrhythmias mechanisms and potential therapies is still needed, but it is difficult to develop a single, universal model for all ventricular arrhythmias mechanisms, as they depend on many biological factors.

## Figures and Tables

**Table 1:** Advantages and disadvantages of animal models used in ventricular arrhythmia research.

Model Type	Advantages	Disadvantages
Pig ischemic	Allows to investigate the impact of coronary artery occlusion on arrhythmia development	Early models with a high degree of invasiveness (sternotomy)
	Enables precise control of ischemia duration and area (balloon catheters)	High animal mortality with LAD occlusion in some protocols
	Facilitates assessment of arrhythmias during both ischemia and reperfusion	Requires complex surgical procedures
	Model with high arrhythmia inducibility	Ischemia–reperfusion model may paradoxically induce arrhythmias during reperfusion
	Translational potential, e.g., studies on the protective effect of spinal cord stimulation (SCS)	Requires specialized equipment (fluoroscopy, balloon catheters, ablation equipment)
	Enables testing of pharmacological and ablative interventions (e.g., PFA)	
	Close similarity to human cardiac physiology	
Pig non-ischemic	Allows to analyze ventricular arrhythmias unrelated to ischemia	Less directly related to human myocardial ischemia pathophysiology
	Enables arrhythmia induction by electrical stimulation (e.g., VF through transthoracic or right ventricular stimulation)	Some models require advanced equipment for stimulation and defibrillation
	Facilitates studies on pharmacological induction of arrhythmias (e.g., bupivacaine)	Less natural reflection of clinical causes of ischemic arrhythmias
	Enables investigation of the impact of epicardial ablation on arrhythmias	Potential limitations in translational relevance to ischemic arrhythmias
	Genetically modified models allow analysis of specific pathological mechanisms Less invasive than ischemic models	
Sheep	Cardiac size and anatomy similar to humans	Different transmural Purkinje fiber system
	Coronary vessels and changes in ventricular muscle after infarction similar to humans	Significant variability in coronary anatomy among individuals
	Develop spontaneous VT/VF	Ethical considerations
	Well-established models of myocardial infarction (ischemic and chronic)	Difficult and expensive to work with
		Different transmural Purkinje fiber system
Canine experimental ischemic models	Close similarity to human cardiac structure and electrophysiology	High invasiveness and surgical complexity
	Biphasic ischemic ventricular arrhythmia pattern. Well-developed Purkinje system enabling reentry and VF	Heterogeneous ischemic protocols
	Controlled ischemia–reperfusion conditions	Advanced surgical and technical requirements
	Strong autonomic modulation enabling neuromodulatory studies	
Canine experimental non-ischemic models	Precise analysis of adrenergic and ionic mechanisms	Reduced representation of clinical ischemic pathology
	High reproducibility of induced arrhythmias	Strong dependence on specific drugs and stimulation protocols
	Suitable for pharmacological testing and VT/TdP induction	
Canine natural models of ventricular arrhythmias	Spontaneous and heritable disease phenotypes	Variable phenotypic expression
	Progressive arrhythmogenic substrates	Limited control over arrhythmia timing
	High incidence of VT, VF, and sudden death	Incomplete genetic characterization in some breeds
	Strong translational relevance to human ARVC and DCM	
Feline experimental ischemic models	Valuable for studying neurogenic modulation of ischemic arrhythmias	Monophasic action potential
	Well-developed sympathetic and parasympathetic control	Limited transmural repolarization gradients
	Suitable for autonomic reflex and baroreceptor studies	Lower susceptibility to reentry-type arrhythmias
Feline experimental non-ischemic models	Insight into neurogenic and anesthetic–adrenergic mechanisms	Strong dependence on anesthesia and experimental conditions
	Useful for pharmacologic and respiratory modulation studies	Ethical limitations in digitalis-based models
	Defined fibrofatty remodeling and ventricular ectopy	Progression toward right-sided heart failure
Feline natural model of ARVC	Rare spontaneous non-canine ARVC model	Small sample size
	Structural and electrophysiological similarity to human disease	No identified genetic mutation

Abbreviations: ARVC—arrhythmogenic right ventricular cardiomyopathy, DCM—dilated cardiomyopathy, LAD—left anterior descending artery, PFA—pulse-field ablation, SCS—spinal cord stimulation, TdP—Torsade de pointes, VF—ventricular fibrillation, VT—ventricular tachycardia.

**Table 2:** Comparative overview of swine models of ventricular arrhythmias and their translational utility.

Species	Model	Arrhythmogenic Mechanism	Methodology	Key Findings	Translational Utility	Limitations	References
Pig	Ischemic model; Open-chest LAD ligation (sternotomy)	Acute ischemia; heterogeneity; VT/VF; Sympathetic-driven ischemic VAs	Sternotomy/Surgical LAD ligation	β-blocker ↓ VF and VT duration/Type Ib arrhythmias linked to ↓ coupling/SCS ↓ ischemic rhythm disturbances	Classic large-animal ischemia arrhythmia platform/Drug testing in acute ischemic VT/VF/Neuromodulation during ischemia	Highly invasive; anesthesia/surgery confounders	[4,5,6]
Pig	Ischemic model; Catheter-based balloon LAD occlusion (I/R)	Ischemia–reperfusion triggers VT/VF	Fluoro-guided balloon LAD occlusion; reperfusion monitoring	Reproducible ischemic vs. reperfusion arrhythmias; survival/inducibility depend on protocol	Clinically analogous I/R model for intervention studies/Neuromodulation in I/R VAs	Cath-lab complexity; site/duration/anesthesia variability; variable survivability	[7,10,11,12,13,14,15]
Pig	Ischemic model; Chronic post-MI scar substrate (balloon LAD)	Scar-related reentry substrate	Prolonged LAD occlusion → reperfusion (weeks) → scar; inducibility testing	Substrate-modifying therapies/ablation reduce inducibility	Translational post-MI VT substrate model	Time/resource intensive; remodeling variability; Specialized equipment	[8,9]
Pig	Ischemic model; Autonomic–neural mechanisms in ischemia/post-MI (mid-LAD balloon ± microspheres; post-MI neural/afferent studies)	Sympatho–vagal imbalance → electrical heterogeneity and VT/VF risk	Mid-LAD balloon ischemia model; post-MI neural/EP phenotyping ± targeted afferent neuromodulation	Sympathetic activation and impaired vagal signaling promote proarrhythmic heterogeneity; afferent targeting can stabilize post-MI hearts	Mechanistic targets for neuromodulation in ischemic cardiomyopathy	Mainly mechanism-oriented; variable endpoints/protocols	[16,17,18]
Pig	Non-ischemic model; Adrenergic VT induction	Triggered activity (catecholamine/cAMP-driven)	Intracoronary/intracardiac NE or cAMP	VT induction without coronary occlusion	Adrenergic triggered-activity model	Limited direct translational relevance; pharmacologic extremum	[19]
Pig	Non-ischemic model; Electrically induced VF	Non-substrate VF trigger	Transthoracic or RV stimulation; resuscitation protocols	Highly reproducible VF induction; both monophasic and biphasic defibrillation are effective in terminating VF	Resuscitation physiology and defibrillation testing	Artificial trigger; not substrate-based	[20,21,22,23]
Pig	Non-ischemic model; PVC-induced cardiomyopathy	PVC burden → remodeling	Chronic pacing to generate frequent PVCs/DCM	Reproducible ectopy-related DCM phenotype	Translational PVC-induced DCM model	Chronic instrumentation; phenotype variability	[24]
Pig	Non-ischemic model; Drug-/procedure-provoked VAs (bupivacaine; epicardial RFA)	Toxicity or iatrogenic triggers	IV bupivacaine; epicardial RFA protocols	VAs with toxicity phenotype; VAs during epicardial ablation safety testing	Toxicology/rescue studies; procedural/device evaluation	Scenario-specific; limited generalizability not substrate-based	[25,26]
Pig	Non-ischemic model; Chronic post-MI + neuromodulation (VNS/TEA)	Autonomic imbalance; reduced inducibility	Chronic MI induction; PES inducibility +/− VNS or TEA	VNS ↓ inducibility (~60%); TEA ↓ inducibility (~70%)	Translational autonomic interventions post-MI	Specialized setup; model complexity	[27,28]

Abbreviations: cAMP—cyclic adenosine monophosphate, DCM—dilated cardiomyopathy, EP—electrophysiology (study), I/R—ischemia–reperfusion, LAD—left anterior descending artery, MI—myocardial infarction, NE—norepinephrine, PVC—premature ventricular complex, RFA—radiofrequency ablation, RV—right ventricle, SCS—spinal cord stimulation, TEA—thoracic epidural anesthesia, VAs—ventricular arrhythmias, VF—ventricular fibrillation, VNS—vagus nerve stimulation, VT—ventricular tachycardia.

**Table 3:** Characteristics of sheep as animal models of ventricular arrhythmias and their translational utility.

Species	Model	Arrhythmogenic Mechanism	Methodology	Key Findings	Translational Utility	Limitations	References
Sheep	Ischemic ventricular arrhythmias	Macro-reentry mechanism involving subepicardial layers at the border of transmural infarction	Dual-site programmed stimulation after myocardial infarction in chronic phase	Reproducible induction of monomorphic VT	Reliable tool to study new techniques for the prevention of ventricular tachyarrhythmias.	Time-consuming method	[36,41]
Sheep	Ischemic ventricular arrhythmias	Reentry mechanism	Dual-site programmed stimulation in early phase after myocardial infarction	Monomorphic sustained VT	In the subacute phase, inducibility of VT and the underlying substrate is predictive of chronic VT.	May not reflect clinical outcomes; in humans, cardiac remodeling may occur for 3 years post-infarct	[40]
Sheep	Bradycardia/ ischemic induced arrhythmias	Reentry mechanism	Atrioventricular node ablation with/without chronic myocardial infarction	PVCs, VT, VF, sudden cardiac death	Model for studying VT and sudden cardiac death.	Bradycardia with myocardial infarction is better than without.	[33]
Sheep	Aconitine induced	Changes in voltage-dependent Na^+^ channels and levels of intracellular Ca^2+^	Aconitine administered into the anterior papillary muscle	VA originating from the papillary muscle, monomorphic VT, focal and stable VT, VF	To investigate mechanisms of degeneration of stable VA into VF in structurally healthy hearts.	The underlying mechanism of degeneration of VA into VF remains unknown	[35]

Abbreviations: PVCs—premature ventricular complexes, VA—ventricular arrhythmia, VF—ventricular fibrillation, VT—ventricular tachycardia.

**Table 4:** Comparative overview of canine ventricular arrhythmia models and their translational utility.

Species	Model	Arrhythmogenic Mechanism	Methodology	Key Findings	Translational Utility	Limitations	References
Dog	Ischemic ventricular arrhythmias (in vivo)	Reentry; ischemia-induced conduction heterogeneity	Coronary artery occlusion; with ECG-based arrhythmia assessment;	Reproducible induction of PVCs, VT, and VF during early ischemia	Gold-standard large-animal model for human ischemic VT/VF and reperfusion arrhythmias	Highly invasive; anesthesia-dependent physiology	[53,54,57,65,66]
Dog	Epinephrine-induced ventricular arrhythmias (in vivo)	Adrenergic overstimulation-induced hypokalaemia and increased myocardial excitability	In vivo epinephrine administration in dogs with assessment of cardiac electrophysiology, blood pressure, and plasma ion concentrations	Excessive adrenergic stimulation promoted hypokalaemia and ventricular arrhythmias, enhanced by environmental exposure	Model reflects human adrenergic arrhythmogenesis due to comparable autonomic responses	Systemic electrolyte effects limit mechanistic specificity	[67,68]
Dog	Torsade do Piontes (QT prolongation-dependent ventricular arrhythmias)	QT prolongation-related early afterdepolarizations and repolarization heterogeneity	In vivo canine models with pharmacologically prolonged QT interval, chronic AV block, programmed stimulation, and epicardial or 3D mapping	TdP induction depended on dynamic repolarization heterogeneity; chronic AV block models showed the highest reproducibility	Clinically relevant model of congenital and drug-induced long QT-associated ventricular arrhythmias	Agent-dependent variability; limited TdP specificity in cesium chloride models	[69,70,71,72,73,74]
Dog—German Shepherd	Inherited ventricular arrhythmias and SCD	Inherited, age-dependent ventricular arrhythmias linked to autonomic imbalance and β-adrenergic hypersensitivity	ECG monitoring and pharmacological β-adrenergic challenge across developmental stages	PVCs, polymorphic VT, and SCD occur during adolescence, often during bradycardia or pauses	Model of inherited ventricular arrhythmias and SCD in structurally normal hearts	Variable phenotypic expression; genetic basis incompletely defined	[75,76,77,78]
Dog—Boxer	Spontaneous ARVC	Desmosomal dysfunction; fibrofatty replacement; conduction discontinuity	ECG, Holter, echo, histopathology, TEM	Frequent PVCs, VT, complex arrhythmias; SCD risk	Best natural large-animal model of human ARVC; highly translatable electrophysiology and pathology	Genetic heterogeneity; variable penetrance	[79,80,81,82,83,84]
Dog—Doberman	DCM with ventricular arrhythmias	Fibrosis; apoptosis; β1-autoantibodies; Ca^2+^ leak	Long-term Holter, echocardiography, biomarker profiling, necropsy	High PVC burden; nsVT/sVT; progression to CHF or SCD	Excellent translational model for risk stratification, biomarker development, and therapy evaluation in human DCM	High mortality; no single causative gene	[85,86,87,88]

Abbreviations: ARVC—arrhythmogenic right ventricular cardiomyopathy, CHF—congestive heart failure, DCM—dilated cardiomyopathy, PVC—premature ventricular complex, nsVT/sVT—non-sustained ventricular tachycardia/sustained ventricular tachycardia, SCD—sudden cardiac death, TdP—Torsade de pointes, TEM—transmission electron microscopy, VF—ventricular fibrillation, VT—ventricular tachycardia.

**Table 5:** Comparative overview of feline ventricular arrhythmia models and their translational utility.

Species	Model	Arrhythmogenic Mechanism	Methodology	Key Findings	Translational Utility	Limitations	References
Cat	Ischemic ventricular arrhythmias (in vivo)	Sympathetic–vagal interplay; neurogenic reflex arrhythmogenesis	LCA occlusion + vagotomy/spinal cord transection	Removal of autonomic input drastically reduces VT/VF incidence	Strong model for studying autonomic contributions to ischemic arrhythmias	Rarely used today; limited scalability	[89]
Cat	Digitalis-induced arrhythmias	Ca^2+^ overload combined with central autonomic modulation	Ouabain administration + CNS lesions (C1 transection, vagal stimulation)	VT triggered via neurogenic pathways; altered toxin threshold	Unique model of neurogenic digitalis toxicity; insights into CNS-cardiac coupling	Strong ethical limitations; outdated clinically	[90]
Cat	Epinephrine-induced arrhythmias under anesthesia	Adrenergic surge; halothane-dependent sensitization; CO_2_ imbalance	Halothane anesthesia + IV epinephrine; ventilation control	Frequent PVCs and VT; hypercapnia exacerbates arrhythmias	Relevant to human perioperative arrhythmias; anesthetic safety research	High dependence on anesthetic agent and ventilation	[91]
Cat—ARVC	Spontaneous ARVC	Fibrofatty myocardial replacement; apoptosis; inflammation	ECG, echo, gross pathology, histology, TUNEL assay	PVCs, polymorphic VT; RBBB; RV aneurysms; apoptosis up to 28%	Rare but powerful natural model mirroring human ARVC pathology and arrhythmogenesis	Small sample (n = 12); CHF predominance; no identified genetic mutation	[92]

Abbreviations: ARVC—arrhythmogenic right ventricular cardiomyopathy, CHF—congestive heart failure, CNS—central nervous system, LCA—left coronary artery, PVC—premature ventricular complex, RBBB—right bundle branch block, RV—right ventricular, VF—ventricular fibrillation, VT—ventricular tachycardia.

## Data Availability

No new data were created or analyzed in this study.
